# Bioactive Glasses Modulate Anticancer Activity and Other Polyphenol-Related Properties of Polyphenol-Loaded PCL/Bioactive Glass Composites

**DOI:** 10.1021/acsami.4c02418

**Published:** 2024-05-06

**Authors:** Michal Dziadek, Kinga Dziadek, Kamila Checinska, Barbara Zagrajczuk, Katarzyna Cholewa-Kowalska

**Affiliations:** † Faculty of Materials Science and Ceramics, Department of Glass Technology and Amorphous Coatings, AGH University of Krakow, 30 Mickiewicza Ave., 30-059 Krakow, Poland; ‡ Faculty of Food Technology, Department of Human Nutrition and Dietetics, University of Agriculture in Krakow, 122 Balicka St., 30-149 Krakow, Poland

**Keywords:** composite, bone substitute, bioactive glass, polyphenols, plant extract, drug delivery, anticancer activity

## Abstract

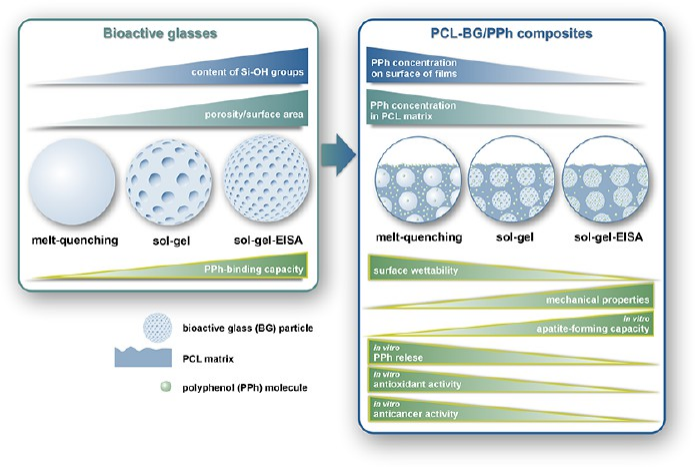

In this work, bioactive glass (BG) particles obtained by three different methods (melt-quenching, sol–gel, and sol–gel-EISA) were used as modifiers of polyphenol-loaded PCL-based composites. The composites were loaded with polyphenolic compounds (PPh) extracted from sage (*Salvia officinalis* L.). It was hypothesized that BG particles, due to their different textural properties (porosity, surface area) and surface chemistry (content of silanol groups), would act as an agent to control the release of polyphenols from PCL/BG composite films and other significant properties associated with and affected by the presence of PPh. The polyphenols improved the hydrophilicity, apatite-forming ability, and mechanical properties of the composites and provided antioxidant and anticancer activity. As the BG particles had different polyphenol-binding capacities, they modulated the kinetics of polyphenol release from the composites and the aforementioned properties to a great extent. Importantly, the PPh-loaded materials exhibited multifaceted and selective anticancer activity, including ROS-mediated cell cycle arrest and apoptosis of osteosarcoma (OS) cells (Saos-2) via Cdk2-, GADD45G-, and caspase-3/7-dependent pathways. The materials showed a cytotoxic and antiproliferative effect on cancerous osteoblasts but not on normal human osteoblasts. These results suggest that the composites have great potential as biomaterials for treating bone defects, particularly following surgical removal of OS tumors.

## 1 Introduction

Osteosarcoma (OS) is the predominant primary bone cancer in children and young adults and ranks second after leukemia as the leading cause of death in young adults.^1^ A combination of pre- and postoperative chemotherapy, along with surgery, is the current standard of treatment for OS. This treatment, resulting in long-term survival rates of over 60% in patients, was formulated in the 1980s. Since then, however, there have been limited advances in treatment strategies.^2,3^ Even after chemotherapy, there is a high risk of recurrence and metastasis, especially to the lungs; other sites in bone, lymph nodes, and other organs; and tissues (e.g., pancreas and skin).^4,5^ In addition, inherent or acquired drug resistance is the main clinical problem that severely limits the success of OS treatment.^2^ High doses of chemotherapy drugs, often used in childhood and adolescent cancers such as OS, cause short and long-term side effects due to their lack of specificity for tumor cells.^1,6^ At the same time, critical-size defects that occur during limb salvage surgery are major concern. Therefore, there is great interest in the development of multifunctional alloplastic bone substitutes with dual osteogenic and selective anticancer activity for the local, safe, and effective treatment of OS.^1,6,7^

One of the most attractive alloplastic bone substitutes are bioactive glasses (BGs).^8,9^ This is primarily due to their ability to bond to living bone, a characteristic attributed to the formation of an apatite layer on the glass surface when exposed to body fluids.^10^ BGs also exhibit osteoinductive properties. Ionic dissolution products (mainly Si and Ca) of BGs at appropriate concentrations have been shown to induce bone regeneration by activating osteogenic genes of osteoblasts and mesenchymal stem cells.^11−13^ Synthesis methods of BGs, including traditional melt-quenching, low-temperature sol–gel, and the relatively novel sol–gel with molecular self-assembly (resulting in so-called template or mesoporous BGs), greatly influence their chemical reactivity and thus the bone-bonding ability and osteogenic response. This is due to the high porosity and surface area and the presence of numerous silanol groups (Si–OH) in the BGs obtained from both sol–gel-based processes. In addition, due to their unique textural properties, mesoporous glasses are being widely considered for use in drug delivery applications.^14−18^

A common application of BG particles is their use as modifiers in biodegradable polymer matrices. An example of this is composites based on poly(ε-caprolactone) (PCL) and BGs. These composites combine excellent biocompatibility, relatively high mechanical strength, ease of processing into various forms (e.g., scaffolds, membranes), and low degradation rate of PCL with excellent bone-bonding ability, osteostimulative properties, stiffness, and high hydrophilicity of BGs.^19−26^

In recent decades, polyphenols (PPh) have been identified as potent anticancer agents. The great interest in polyphenols is due to their multifaceted antitumor activity, including modulation of pathways associated with cell proliferation, migration, angiogenesis, metastasis, and cell death.^27^ Importantly, their selective activity may minimize the side effects of traditional cancer treatments (radiotherapy, chemotherapy) by specifically targeting cancer cells without affecting normal cells.^28^ Polyphenols have also been shown to improve the sensitivity of cancer cells, including OS and multidrug-resistant cells, to chemotherapy.^29^

Among the many different types of polyphenols, those derived from sage (*Salvia officinalis* L.) are already being considered in cancer treatment. Sage extracts have been reported to have high anticancer activity in a number of different cancer cells.^30−32^ Furthermore, there is ample evidence that rosmarinic acid, carnosic acid, and carnosol, the main polyphenolic compounds (PPh) of sage extracts (also extract used in this study^33^), have potent anticancer activity against many types of cancer cells, including OS.^5,28,34,35^

In previous work, we have demonstrated the synergistically enhanced effect of the combination of sol–gel-derived BG (designated as A2) and PPh extracted from sage in PCL-based composites on osteogenic osteoblast response. In addition, the polyphenols improved the apatite-forming ability of the composites, while providing antioxidant and anti-inflammatory activity and antibiofilm properties against Gram-positive and Gram-negative bacteria.^33^

In this work, BG particles with the same composition (also A2) and obtained by three different methods (melt-quenching, sol–gel, and sol–gel-EISA) were used as modifiers of polyphenol-loaded PCL-based composites. The composites were loaded with PPh extracted from sage (*Salvia officinalis* L.). We expected that the polyphenols would provide the composites with selective anticancer activity against OS cells. This, together with their osteogenic, anti-inflammatory, and antibiofilm properties, could result in multifunctional bone substitutes for the treatment of bone defects, especially after tumor resection. Furthermore, we hypothesized that BG particles would bind the polyphenols to different extents due to their different textural properties (porosity, surface area) and surface chemistry (content of silanol groups). This in turn modulates the release of polyphenols from PCL/BG composites and other significant properties, associated with (antioxidant and anticancer activity) and affected by (wettability, mechanical properties, and apatite-forming ability) the presence of PPh.

## 2 Materials and Methods

### 2.1 Material Preparation

According to previous work, polyphenols were obtained from lyophilized leaves of sage (*Salvia officinalis* L.). They were extracted in a mixture of 1,4-dioxane and water. The extract consists mainly of phenolic acids (mainly rosmarinic acid), flavonoids (mainly epicatechin), and phenolic diterpenes (carnosol and carnosic acid).^33^

The melt-quenching, sol–gel, and sol–gel with evaporation-induced self-assembly (EISA) techniques were used to synthesize BGs (A2, 40SiO_2_–54CaO–6P_2_O_5_ mol %), as described previously.^36−39^ The BGs were designated as A2mq, A2sg, and A2sa, respectively. BG powders with a particle size of 1.5 μm (*d*_50_) were obtained by milling. BGs were analyzed using SEM, EDX, TEM, FTIR, and BET methods according to our previous works.^38,39^ The results are presented in the Supporting Information.

The PCL-based films were produced using the solvent casting method according to our previous work.^33^ The composite contained 30% w/w BG and 4.5% w/w PPh. Furthermore, for in vitro cell studies, materials containing 1.5% w/w of PPh were fabricated (Table 1). The surface of the films that were exposed to the solvent vapor during the casting process was designated as AS, while the surfaces that were in contact with the glass Petri dish were marked as GS.

**Table 1:** Compositions of Materials

material	BG synthesis method	BG (% w/w)	PPh (% w/w)
PCL			
PCL/15PPh			1.5
PCL/45PPh			4.5
PCL-A2mq	melt-quenching	30	
PCL-A2mq/15PPh	melt-quenching	30	1.5
PCL-A2mq/45PPh	melt-quenching	30	4.5
PCL-A2sg	sol–gel	30	
PCL-A2sg/15PPh	sol–gel	30	1.5
PCL-A2sg/45PPh	sol–gel	30	4.5
PCL-A2sa	sol–gel-EISA	30	
PCL-A2sa/15PPh	sol–gel-EISA	30	1.5
PCL-A2sa/45PPh	sol–gel-EISA	30	4.5

### 2.2 Material Evaluation

#### 2.2.1 Evaluation of the Surface Properties

The ultrahigh resolution scanning electron microscope (SEM) was employed to assess the microstructure and chemical composition of the films. The SEM was equipped with a field emission gun and a secondary electron detector (Nova NanoSEM 200 FEI Europe Company, accelerating voltage 10–18 kV) coupled with an energy dispersion X-ray (EDX) analyzer with a SiLi detector (EDAX, the Netherlands). To assess the samples using SEM, they were coated with a carbon layer. The sessile drop method was employed to evaluate surface wettability and solid-state surface energy (SE) by using the automatic drop shape analysis system DSA 25 (Kruss, Germany). The Owens-Wendt-Rabel-Kaelble (OWRK) method was used to calculate SE. The following liquids were employed: ultrahigh-quality water (UHQ, PureLab, Vivendi Water) and diiodomethane (Sigma-Aldrich, Germany). Both parameters were calculated by averaging 10 measurements. The results were expressed as the mean ± standard deviation (SD). Both surfaces of the films (AS and GS) were subjected to analysis.

#### 2.2.2 Evaluation of Mechanical Properties

The universal testing machine Inspect Table Blue 5 kN (Hegewald&Peschke, Germany) was used to determine tensile strength (σ_M_), Young’s modulus (*E*_*t*_), and elongation at maximum force (ε_M_). The machine was equipped with a 100 N load cell. The samples for mechanical testing were rectangular in shape (30 × 5 mm). The tests were performed with the preload force of 0.1 N and the test speed of 10 mm min^–1^. Mechanical properties were calculated by averaging 10 measurements and were expressed as the mean ± SD.

#### 2.2.3 In Vitro Apatite-Forming Ability Test

Incubation of the materials in simulated body fluid (SBF) was used to evaluate their apatite-forming ability.^40^ The incubation was performed at 37 °C for the periods of 3, 7, and 14 days, as described previously.^33^ The sample weight to SBF volume ratio was 10^–3^ g mL^–1^.^22^ SEM/EDX, FTIR, and ICP-OES methods were employed to evaluate the samples and SBF, respectively, according to our previous work.^33^ The measurements of Ca, P, and Si concentrations in the SBF were performed in triplicate and expressed as the mean ± SD.

#### 2.2.4 In Vitro Polyphenol Release and Antioxidant Activity Testing

The PPh-containing films were incubated in phosphate-buffered saline (PBS; pH = 7.4, HyClone, USA) at 37 °C for 120 h. The sample weight to medium volume ratio was 8 × 10^–3^ g mL^–1^. At specified intervals, PBS was sampled and replaced with the same volume of fresh PBS. PPh concentration in PBS was evaluated using POLARstar Omega microplate reader (BMG Labtech, Germany) at 320 nm, according to our previous work.^33^ The percentage of released PPh was determined in relation to the initial PPh content incorporated into the materials.

ABTS and DPPH free radical scavenging assays and ferric-reducing antioxidant power (FRAP) methods were used to evaluate antioxidant activity of the materials, according to our previous work.^33^ The radical scavenging capacity (RSC) of the materials was calculated as follows: RSC = (*A*_0_ – *A*_S_/*A*_0_), where *A*_S_ was the absorbance of the solution after sample incubation and *A*_0_ was the absorbance of ABTS and DPPH working solutions. FRAP results were expressed as absorbance. Polyphenol release and antioxidant activity tests were performed in triplicate and expressed as the mean ± SD.

#### 2.2.5 In Vitro Cell Studies

The normal human osteoblasts (NHOst, Lozna, USA) and Saos-2 human OS cells (ATCC, USA) were expanded in Nunclon Delta 75 cm^2^ culture flasks (Nunc, Denmark) and cultured under standard conditions in a humidified atmosphere containing 5% CO_2_, at 37 °C. NHOst cells were cultured in OBM Basal medium, supplemented with OGM Osteoblast Growth BulletKit (10% FBS, 1% ascorbic acid, and 1% gentamicin, Lozna, USA). The Saos-2 cells were cultured in McCoy 5A medium (Gibco, USA), supplemented with 15% FBS (Gibco, USA). The culture medium was changed every 3 days. Cells were detached from culture flasks using trypsin/EDTA solution (Gibco, USA) with 75% (NHOst) and 85% (Saos-2) confluence. Cells were seeded into wells of 48-well culture plates (Nunc, Denmark) in direct contact with biomaterial samples in a density of 5 × 10^4^ cells/mL/well and cultured for 1, 3, and 5 days in standard culture conditions. To prevent the samples from floating, ultrapure silica glass inserts (Continental Trade, Poland) were used. The GS surface of the films was used to culture the cells.^23^ The samples were sterilized by exposure to UV–C light (each surface for 15 min) and subsequently washed with sterile PBS (HyClone, USA).

The cell proliferation and cytotoxicity of the materials were assessed using a ToxiLight BioAssay Kit and ToxiLight 100% Lysis Reagent Set (Lonza, USA) according to the manufacturer protocol. The supernatant was employed to determine the number of damaged cells, while the lysate was used to identify the number of intact adherent cells. The luminescence was measured with an Omega POLARstar Microplate Reader (BMG Labtech, Germany).

The cell-permeable fluorogenic probe DCFH-DA (Sigma-Aldrich, USA) was used to measure intracellular reactive oxygen species (ROS) production. The cells were washed with PBS and incubated in 400 μL of l0 μM DCFH-DA-PBS solution for 30 min at 37 °C in a CO_2_ incubator. The fluorescence was measured with an Omega POLARstar Microplate Reader (BMG Labtech, Germany).

Growth arrest and DNA damage-inducible protein gamma (GADD45G) concentration was measured with a GADD45G ELISA Kit (AssayGenie, Ireland) according to manufacturer protocol. The absorbance was measured with an Omega POLARstar Microplate Reader (BMG Labtech, Germany).

Cyclin-dependent kinase 2 phosphorylated at Tyr15 (phospho-CDK2 (Tyr15)) concentration was measured with an ElisaCell Cycle In-Cell ELISA Kit (Abcam, UK) according to manufacturer protocol. The fluorescence was measured with an Omega POLARstar Microplate Reader (BMG Labtech, Germany).

Caspase-3/7 activity was measured with Caspase-Glo 3/7 Assay (Promega, USA) according to manufacturer protocol. The luminescence was measured with an Omega POLARstar Microplate Reader (BMG Labtech, Germany).

All of the results were normalized to the number of intact adherent cells. The results were presented as the mean ± SD and represented at least four independent experiments.

### 2.3 Statistical Analysis

The results were analyzed using one-way analysis of variance (ANOVA) with Duncan post hoc tests, which were performed with Statistica 13 (StatSoft, USA) software. The results were considered statistically significant when *p* < 0.05.

## 3 Results and Discussion

Previous studies have shown that polyphenols, both individual (e.g., gallic acid) and extracted from plants (e.g., green tea leaves, red grape skin, and dog rose buds), can readily bind to the surface of BGs and glass-ceramics in bulk and powder form. These interactions are enabled by the formation of hydrogen bonds between the abundant hydroxyl groups of PPh and the silanol groups of BGs. All of the materials tested were traditional melt-derived ones that required surface activation to couple with the polyphenols. Surface activation was achieved by exposing the reactive hydroxyl groups through simple water washing.^7,41−47^ Zhang et al. demonstrated that polyphenol-binding capacity of melt-derived BGs increased with the glass reactivity (apatite-forming ability). The differences in the reactivity of the glasses directly resulted from their different composition.^44,45^

In some papers, the authors have shown that the presence of polyphenol-binding fillers in the polymer matrix can modulate polyphenol release kinetics and polyphenol-related properties. Shao et al. demonstrated that the introduction of multiwalled carbon nanotubes (MWCNTs) into PCL-based electrospun nanofibers reduced the concentration of green tea polyphenols (GTPs) in the polymer matrix of nanofibers and on their surface. The authors have suggested that this phenomenon can be explained by the adsorption of GTPs onto surface of the MWCNTs through π–π interactions between nanotubes and the GTP hydroxyl groups.^48^ Monavari et al. shown that sol–gel-derived mesoporous silica-calcium nanoparticles (MSNs) can adsorb icariin while modulating its release profile and kinetics from 3D-printed alginate dialdehyde-gelatin/MSNs hydrogels, and thus the behavior of MC3T3-E1 preosteoblasts. The high icariin-loading capacity of MSNs was attributed to the presence of silanol groups on their surface and the mesoporous structure.^49^ In our recent works, we have shown that the sol–gel-derived BG (A2) particles would serve as an agent for controlling the release of the sage-derived PPh from PCL/BGs composite films and other important properties related to and influenced by the presence of polyphenols (e.g., long-term degradation, mechanical properties, wettability, in vitro apatite-forming ability, antioxidant activity, cytotoxicity, and in vitro osteogenic and anti-inflammatory properties).^33,50^

In this work, three BGs with the same composition (also A2) and synthesized with different methods (melt-quenching, sol–gel, and sol–gel with EISA) were used to obtain PCL-based composite films loaded with polyphenols extracted from sage. The results of the polyphenol release kinetics from the composites as well as other properties discussed below indicated that these fillers had very different polyphenol-binding capacities. This can be attributed to the significant differences in the textural properties (porosity, surface area, Figure S1, Table S1) and surface chemistry, namely, the content of silanol groups, of the BGs (Figure S2). The higher the porosity and the surface area of the BGs as well as the content of silanol groups, the higher the expected binding capacity. It should be mentioned that during the preparation procedure of the composites, BG particles were contacted with the plant extract for 24 h prior to the addition of PCL to allow PPh to bind to the fillers. Importantly, melt-derived BG was not activated before contacting with PPh. However, because of the high reactivity of A2 BGs resulting from the high calcium oxide content in the glass composition (54 mol %), the formation of hydroxyl groups on the surface of the melt-derived glass occurred in contact with moisture from the air during glass handling (Figure S2). In addition, exposure of the glass to the water-containing solvent mixture (1,4-dioxane/deionized water mixture with a volume ratio of 4:0.3) can promote this process. Therefore, reactivity and thus binding capacity of BGs can be ordered as follows: A2mq (low S_A_, low silanol content) < A2sg (high S_A_, high silanol content) < A2sa (very high S_A_, very high silanol content).

**Figure 1:**
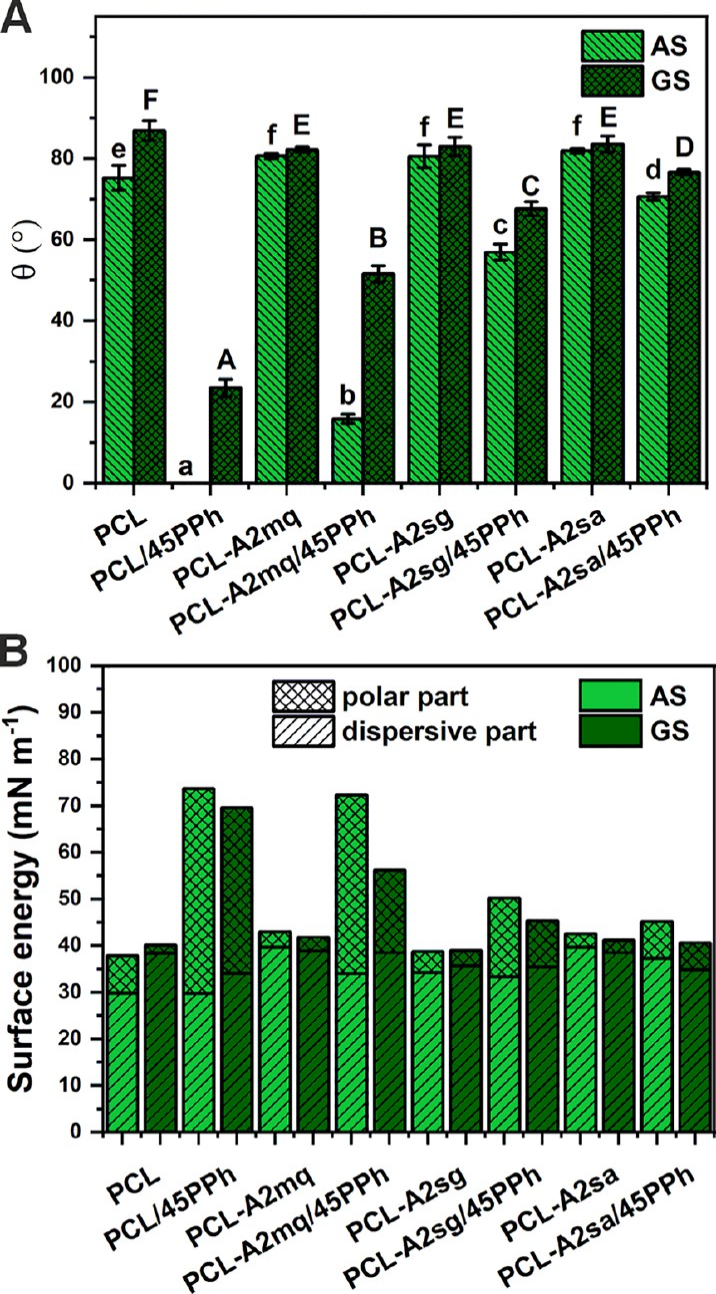
Static water contact angle (A) and total SE (B) with its dispersive and polar components of both surfaces (AS and GS) of the films. Statistically significant differences (*p* < 0.05) for AS and GS are indicated by subsequent lower and upper Latin letters, respectively. Different letters indicate statistically significant differences.

Figure 1 shows the results of the static water contact angle (θ) and surface energy (SE) of AS and GS of the films. AS of the PCL film showed higher hydrophilicity compared to its GS. The presence of BG particles, regardless of type, significantly increased AS contact angle values, whereas those for GS were much lower. This resulted in similar wettability of both surfaces of the composites without PPh. The wettability of both surfaces was significantly improved by the incorporation of PPh into all films. However, the effect was particularly pronounced on AS, resulting in much greater differences in contact angle values between AS and GS. The changes depended on the modification used. The most wettable was the PCL/PPh film. For the PPh-modified composites films, wettability decreased in the following order: PCL-A2mq/45PPh > PCL-A2sg/45PPh > PCL-A2sa/45PPh.

The incorporation of PPh into the films led to a notable increase in the total SE of both surfaces (Figure 1B). The main reason for this was an increase in the value of the polar component with a smaller effect of a decrease in the value of the dispersive component. This correlated with changes in wettability. The more wettable the PPh-modified materials, the higher the value of the total SE and its polar component.

The significantly increased polar part of the SE along with improved hydrophilicity can be attributed to the hydroxyl groups of PPh exposed on the surfaces of the films. This was supported by results obtained by Kim et al.^51^ for the electrospun PCL nanofibers modified with phlorotannin. The differences observed between the AS and GS of PPh-loaded films can be attributed to the varying concentrations of PPh present on the respective surfaces. A higher concentration of PPh on AS, and therefore greater wettability, can be attributed to the high mobility in solution as a result of their relatively low molecular weight. As solvent molecules migrate toward the AS surface of the film during evaporation, they simultaneously facilitate the movement of PPh molecules in the same direction. Our previous research using FTIR analysis confirmed a higher concentration of PPh on the AS of the PCL/PPh-based films.^33^ There was a direct correlation between the polyphenol-binding capacity of the BGs and the surface wettability of the composites. Indeed, the higher the polyphenol-binding capacity of the BGs, the lower the amount of polyphenols on the surface of the composite and therefore the lower the improvement in hydrophilicity.

**Figure 2:**
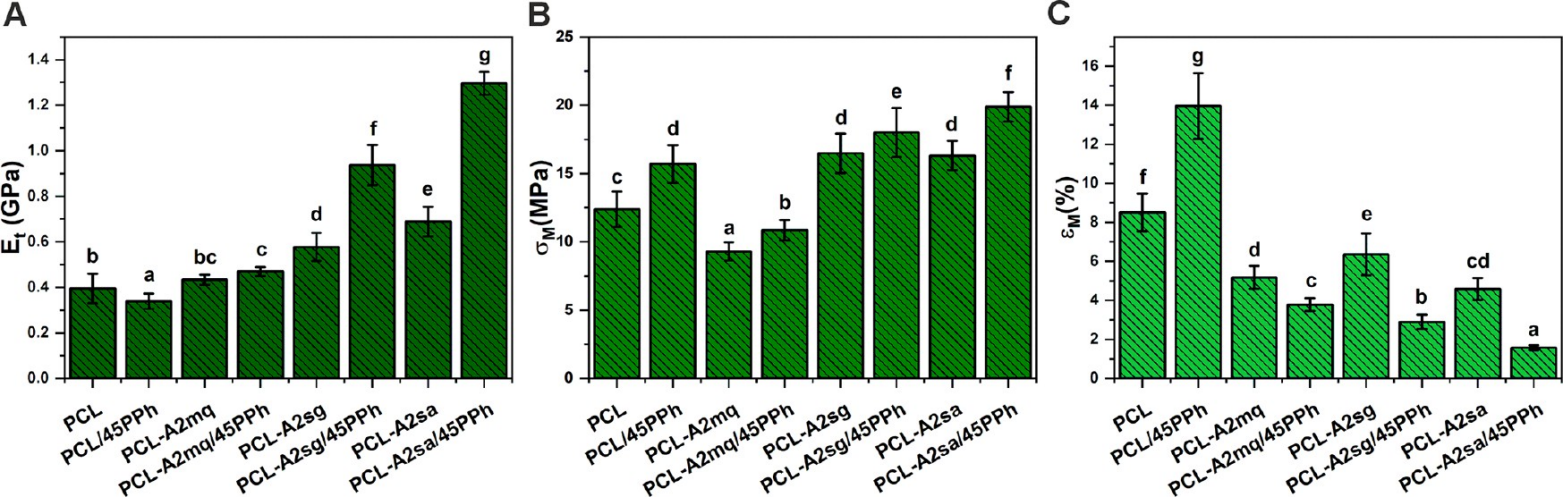
Young’s modulus (A), tensile strength (B), and elongation at maximum force (C) of the films. Statistically significant differences (*p* < 0.05) are indicated by subsequent lower Latin letters. Different letters indicate statistically significant differences.

Figure 2 shows the results of Young’s modulus (*E*_*t*_), tensile strength (σ_M_), and elongation at maximum force (ε_M_) of the films. The presence of sol–gel- and EISA-derived BGs in the PCL matrix significantly improved both *E_t_* and σ_M_. A greater increase in the *E_t_* was observed in the composite with EISA-derived BG (PCL-A2sa). Between these two composites, the values of σ_M_ were not significantly different. For the composite modified with melt-derived BG (PCL-A2mq), *E*_*t*_ did not change significantly, while σ_M_ decreased. This was accompanied by a decrease in the ε_M_ for all composites. Modification of the PCL film with PPh resulted in a reduction of *E_t_*, while σ_M_ and ε_M_ increased significantly. In general, the PPh-modified composites showed significantly improved *E_t_* and σ_M_ as well as reduced ε_M_ in comparison with materials without PPh. The only exception was PCL-A2mq/45PPh film, for which the *E*_*t*_ value did not change significantly when compared to the composite without PPh. The greatest increase in the values of both parameters when introducing PPh was observed in composites modified with EISA-derived glass (PCL-A2sa/45PPh). Remarkably, the incorporation of both EISA-derived BG particles and PPh enabled *E*_*t*_ values almost four times higher than those observed with the PCL/PPh film to be achieved. As we have shown in our previous work, polyphenols can interact not only with BGs but also with PCL.^33^ Hydrogen bonds can easily form between carbonyl groups of PCL and hydroxyl groups of PPh.^52,53^ This can have a two-way effect on the mechanical properties of the films. First, as the PCL/PPh material showed a significant reduction in *E_t_* and an increase in ε_M_ compared to the PCL film, PPh can be considered as a green plasticizer for PCL. The decrease in melting temperature and the increase in crystallinity and crystallite size of PCL upon modification with PPh have been observed in our previous work, suggesting that PCL chains interact with polyphenolic molecules.^33^ Second, PPh can act as a coupling agent, providing linkages between the fillers and the polymer matrix in the composite films. This was reflected in significantly improved mechanical performance of PPh-modified composites, particularly *E*_*t*_ and σ_M_, compared to the films without PPh. Again, a strong correlation was observed between the PPh-binding capacity of BGs and improvement of the mechanical properties of the composite films. The presence of BG with a higher PPh-binding capacity in the polymer matrix resulted in a greater increase in *E*_t_. This may be due to improved interaction at the PCL-BG interface and a reduced plasticizing effect on the PCL matrix.

**Figure 3:**
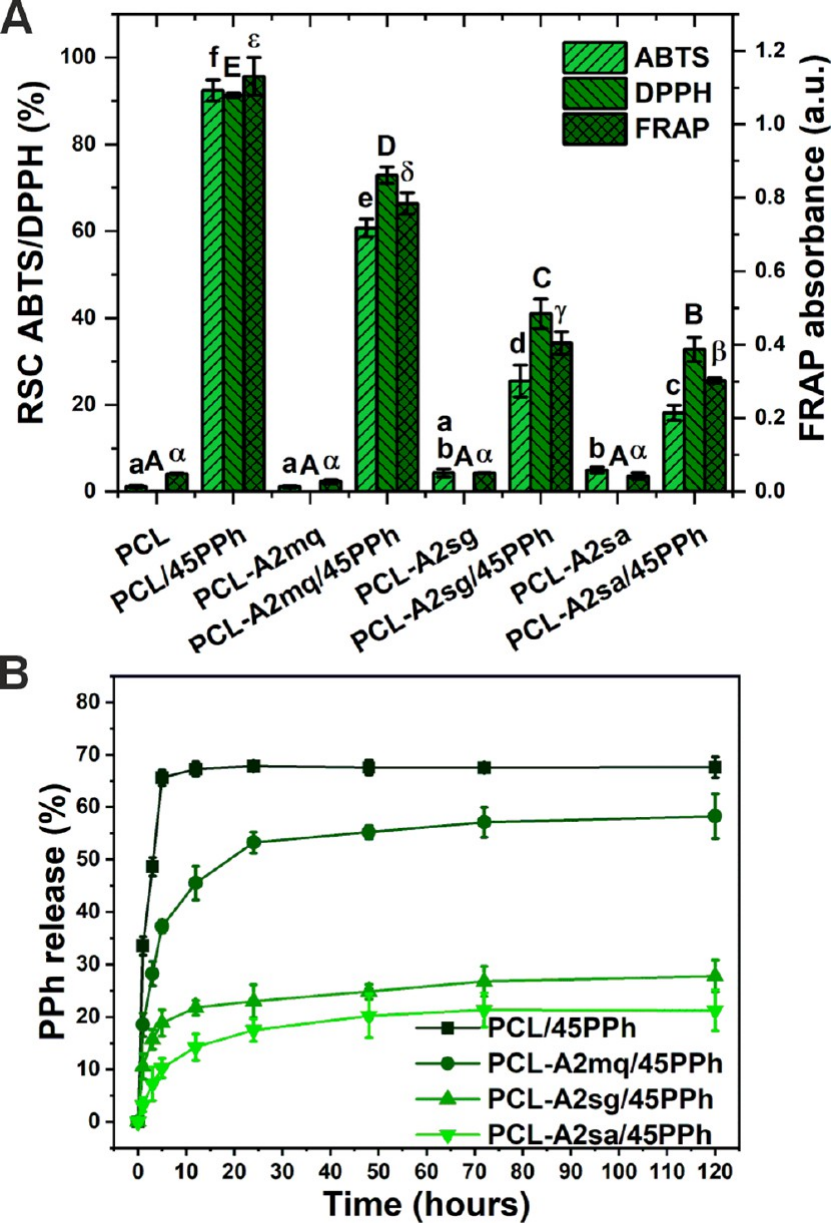
Radical scavenging capacity (RSC) against the ABTS^**·**+^ and DPPH**^·^** radicals as well as ferric-reducing antioxidant potential (FRAP) of the films (A). Statistically significant differences (*p* < 0.05) are indicated by subsequent lower, upper Latin letters and Greek letters, respectively. Different letters indicate statistically significant differences. The release profiles of polyphenols presented as percentage of released PPh in reference to the initial content in the films (B).

Figure 3A shows the RSC against the ABTS^**·**+^ and DPPH**^·^** radicals as well as the FRAP of the films. In materials without PPh, virtually no antioxidant activity was observed. Films containing PPh showed significantly higher RSC and reducing potential, although these were strongly dependent on the presence and type of the BGs. The material with the highest antioxidant activity was found to be PCL/PPh. For the PPh-modified composite films, RSC and reducing potential decreased in the following order: PCL-A2mq/45PPh > PCL-A2sg/45PPh > PCL-A2sa/45PPh.

The release profiles of PPh from the films are presented in Figure 3B. Within the first few hours, the materials showed a burst release of PPh. However, the presence of BG particles reduced the rate of release. For the PCL-based materials, the maximum concentration of PPh in the medium was reached after 5 h, whereas for the composites, the release was slower, and the maximum values were reached after 72 h. In addition, the percentage of PPh that was released was dependent on the type of BGs. The PCL/PPh material achieved the highest level of release (68%). The composites showed lower levels of release in the following decreasing order: PCL-A2mq/45PPh (58%) > PCL-A2sg/45PPh (28%)>PCL-A2sa/45PPh (21%).

The results clearly showed that by using fillers in the form of BGs with different textural properties and surface chemistry, both the antioxidant activity and the kinetics of polyphenol release from PCL-based materials can be modulated. BGs that bind PPh more effectively reduced the overall release level and burst effect. This was directly related to the reduced concentration of PPh in the polymer matrix of the films and on their surface. Shao et al. found a similar delay in the release of GTPs after modification of the PCL matrix with MWCNTs capable of binding polyphenols.^48^ The polyphenols both released from the material and bound to its surface are responsible for the RSC and reducing potential. Therefore, the same correlations were observed between the PPh-binding capacity of the BGs and the antioxidant properties of the films.

**Figure 4:**
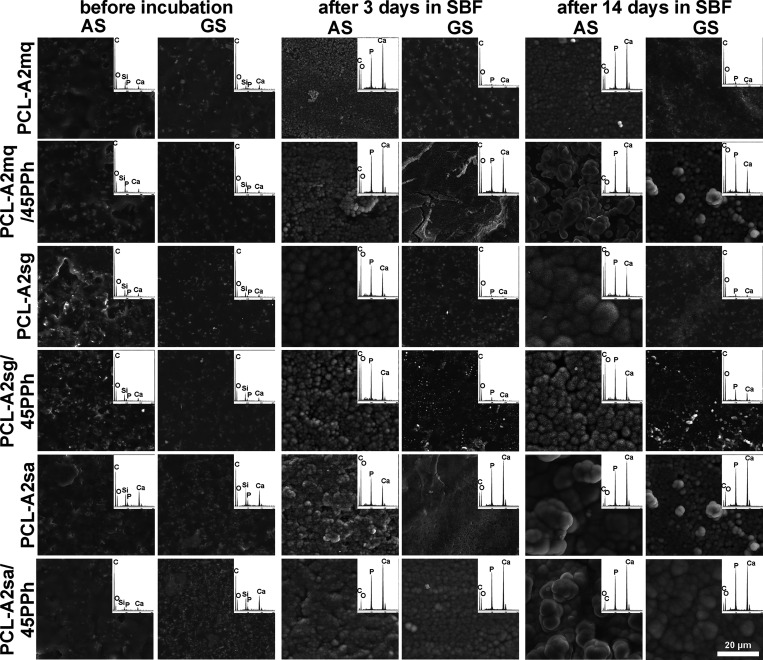
SEM images and EDX spectra (averaged for the entire analyzed surface) of the AS and GS of the films before and after 3- and 14-day incubation in SBF.

Figure 4 shows SEM images and EDX spectra of GS and AS of the films before and after incubation in SBF. Even after 14 days of incubation, no chemical or morphological changes were observed on the surfaces of either PCL or PCL/PPh films (data not shown). After 3 and 14 days, the formation of a calcium phosphate layer was observed on the surfaces of the composite films. However, the morphology and concentration of Ca and P differed among the materials and surfaces tested. The AS of all composite films exhibited the formation of spherical, cauliflower-like crystals rich in Ca and P, characteristic of carbonated hydroxyapatite (HCA). These were observed after only 3 days of incubation. The GS of PCL-A2mq/45PPh, PCL-A2sa, and PCL-A2sa/45PPh films were also covered with a layer with HCA morphology and high Ca and P concentrations but generally finer in size. In both cases, the crystals became larger after a longer incubation period. In contrast, the layer formed on GS of PCL-A2mq, PCL-A2sg, and PCL-A2sg/45PPh after 3 and 14 days of incubation did not show a typical HCA morphology and exhibited lower Ca and P concentrations. The absence of Si from the BGs after incubation indicated that the layers were thick and covered both surfaces of all composite films homogeneously.

**Figure 5:**
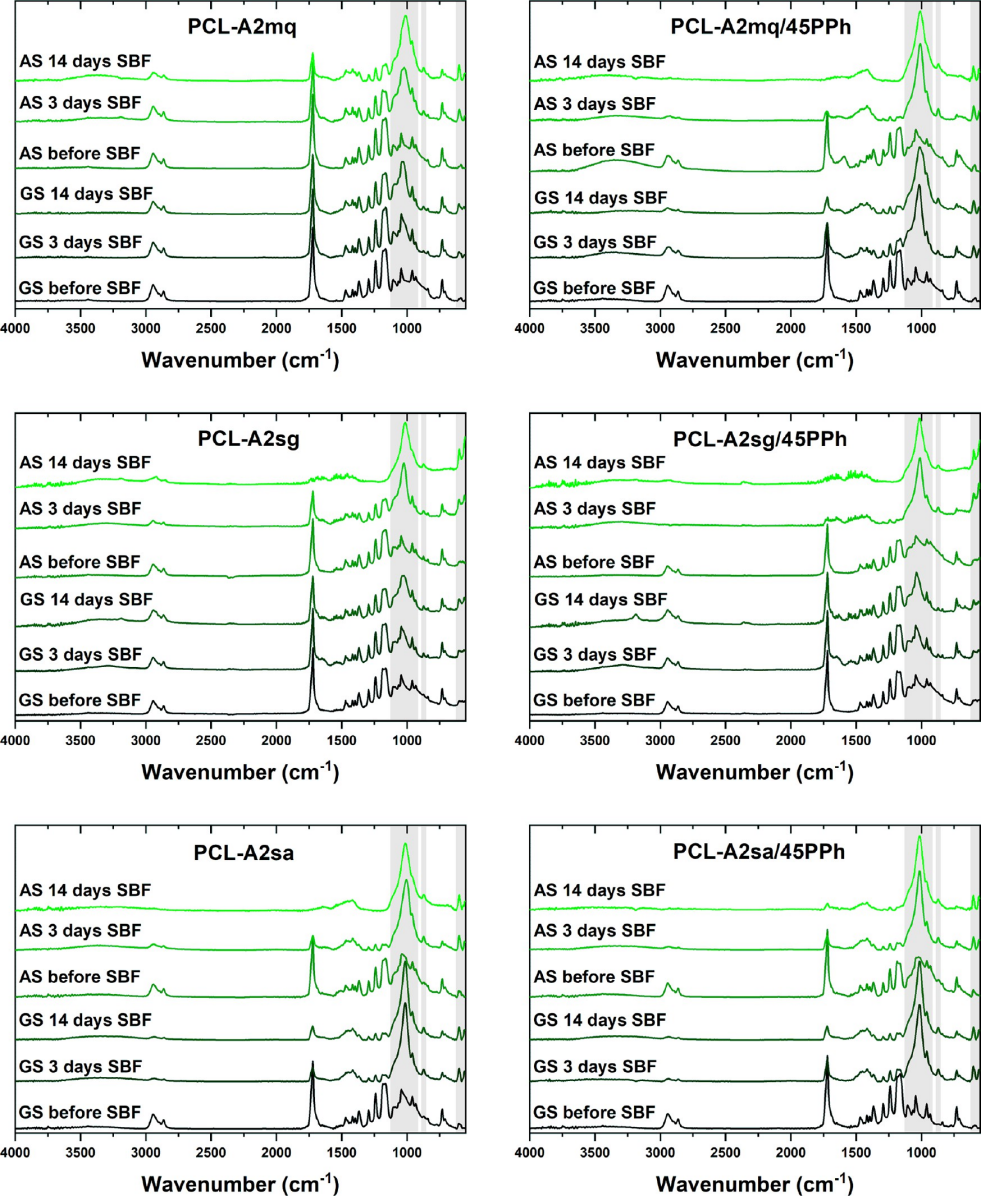
ATR-FTIR spectra of AS and GS of the films before and after 3- and 14-day incubation in SBF.

Figure 5 shows the ATR-FTIR spectra of GS and AS of the films before and after incubation in SBF. The PCL and PCL/PPh films did not show any significant changes during the incubation (data not shown). For all composites, incubation for 3 days resulted in the appearance of new bands in the 560–602 cm^–1^ and 910–1180 cm^–1^ regions. They can be attributed to the bending and stretching modes, respectively. The band at 875 cm^–1^ could be assigned to the P–O stretching mode of the HPO_4_^2–^ ions and/or to the out-of-plane bending mode of the CO_3_^2–^ ions. These new bands are characteristic of nanocrystalline nonstoichiometric apatite.^54^ The apatite bands exhibited a progressive increase in intensity, while the PCL bands exhibited a corresponding decrease, indicating that the thickness of the layer increased with increasing incubation time. Nevertheless, a markedly more rapid change in band intensity was observed in the case of AS compared to GS. Faster changes were also observed for PCL-A2mq/45PPh, PCL-A2sa, and PCL-A2sa/45PPh films. This confirmed the accelerated and more effective formation of the apatite layer for AS and the abovementioned films.

**Figure 6:**
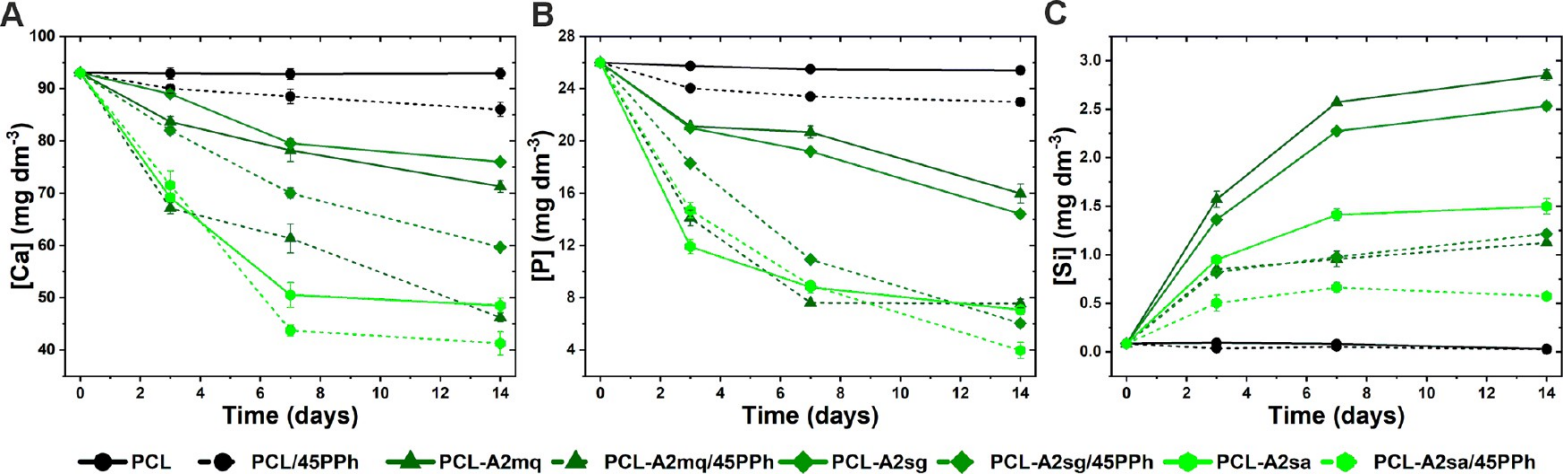
Changes of Ca (A), P (B), and Si (C) concentrations in SBF during incubation of the films.

Figure 6A,C shows the results of the ICP-OES analysis of the concentrations of Ca, P, and Si in SBF during film incubation. The concentrations of individual elements in the SBF remained unchanged for the PCL film. For the PCL film containing PPh (PCL/45PPh), a slight reduction in Ca and P was observed. In contrast, the concentrations of Ca and P showed a gradual decrease for the composites. However, the composites modified with different BGs and those containing PPh showed different rates of change. The composite containing A2sa (PCL-A2sa) showed a significantly faster reduction in Ca and P concentrations compared to films with A2mq (PCL-A2mq) and A2sg (PCL-A2sg), for which the change profiles were similar. The presence of PPh in composite films accelerated the Ca and P depletion. Interestingly, PCL-A2mq/45PPh showed significantly greater changes than PCL-A2sg/45PPh, although the behavior of these composites without PPh was similar. Conversely, the differences between the rates of change in Ca and P concentrations for the PCL-A2sa and PCL-A2sa/45PPh composites were not as pronounced. These results were consistent with changes in the concentration of Si in the SBF. In general, Si release was lower in composites with greater decreases in Ca and P. This was due to the faster and more effective formation of the apatite layer, which slowed the solubility of Si. For the same reason, after 7 days of incubation, a significant decrease in the rate of Si release from the materials was observed.

The results of SEM-EDX, FTIR, and ICP-OES analyses clearly showed that polyphenols promote and accelerate the formation of the apatite layer on BG-containing films. These results are consistent with those previously observed by the authors for PCL/BG films modified with polyphenols.^33^ The beneficial effect of polyphenols on the apatite-forming capacity of silicate BGs grafted with polyphenols extracted from plants (green tea leaves, red grape skin, and dog rose buds) has also been observed in previous works.^42,46^ This may be due to the presence of polyphenols on the surface of the materials, which expose numerous hydroxyl groups. These groups can interact with calcium ions and serve as nucleation centers for apatite crystallization.^55,56^ In addition, the enhanced ability to form an apatite layer may be related to the improved wettability and higher SE, particularly its polar component, of the PPh-modified films.^57^ The following results support these speculations. First, the ability of the PCL/45PPh film to adsorb calcium and phosphorus from SBF is in comparison to the unmodified PCL film. Second, when comparing materials with and without PPh, the greatest improvement in apatite-forming ability by PPh in films modified with A2mq. As shown in previous results, A2mq BG binds PPh the least, resulting in the highest concentration of PPh on the surface of the composite. Importantly, although A2sa BG binds polyphenols most efficiently, the PCL-A2sa/45PPh film shows a high apatite-forming capacity. In this case, this was due to the high reactivity of the A2sa BG itself, which was confirmed by the results for the film without PPh (PCL-A2sa).

For the in vitro cell test, based on our previous studies on PCL-A2sg/PPh-based composites, the concentration of 1.5% w/w of PPh in the films was chosen. It is important to note that higher concentrations of PPh (3 and 4.5% w/w) in the composites have been found to be cytotoxic in static in vitro cell culture.^33^

**Figure 7:**
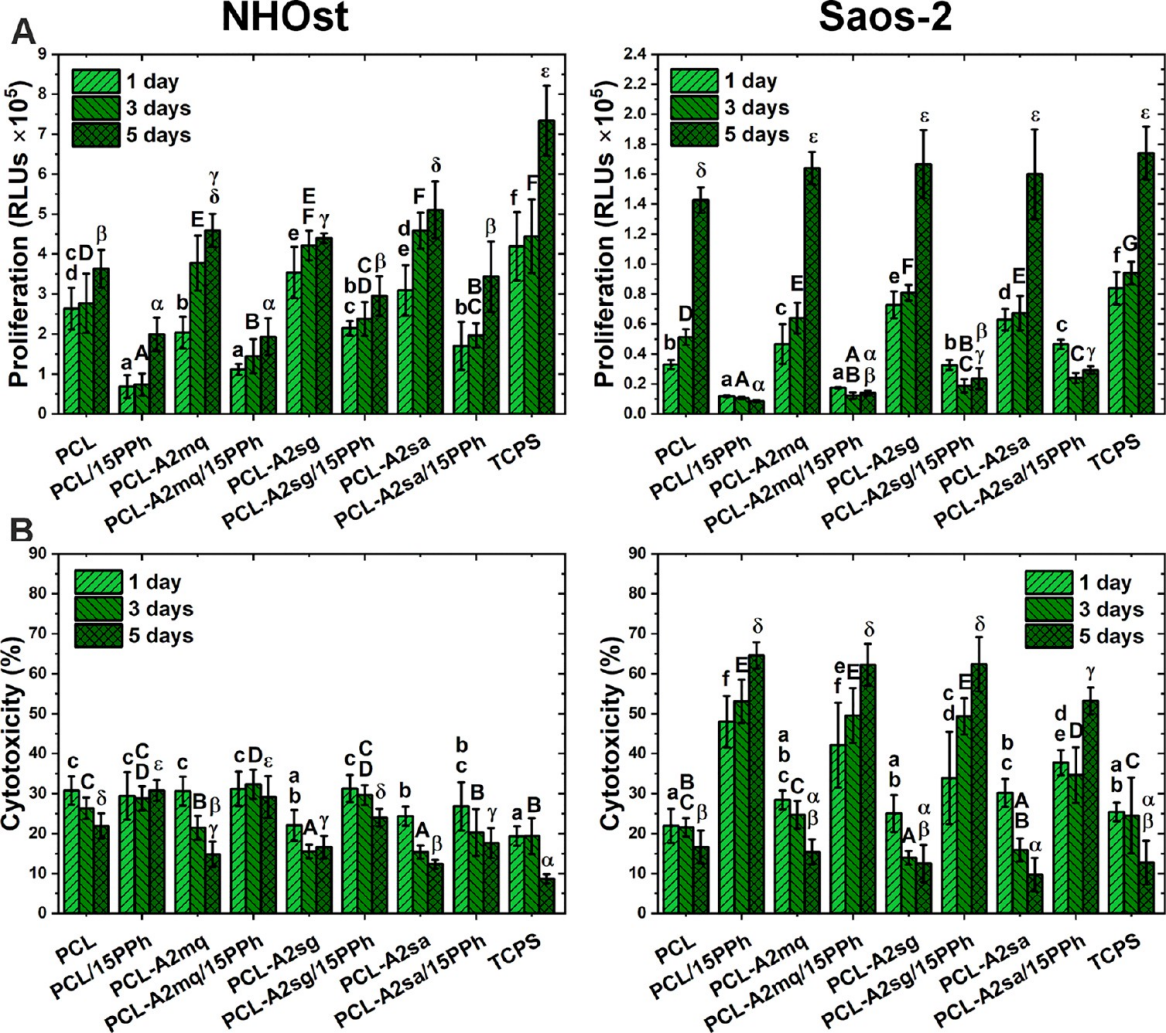
Adenylate kinase (AK) level in the lysate corresponding to the number of intact adherent cells (A) and AK level in the supernatant related to AK level in the lysate representing material cytotoxicity (B). Statistically significant differences (*p* < 0.05) between films and TCPS after 1-, 3-, and 5-day cell culture periods are indicated by subsequent lower, upper Latin letters and Greek letters, respectively. Different letters indicate statistically significant differences.

The proliferation of both normal (NHOst primary osteoblasts) and cancerous (Saos-2 OS cell line) human osteoblasts cultured in direct contact with the films and the cytotoxicity of the materials is shown in Figure 7A,B, respectively. The number of NHOst increased over the culture period for all films, indicating that the materials supported the proliferation of normal osteoblasts. On the other hand, while the materials without PPh also supported the proliferation of cancer cells, the number of Saos-2 cells cultured on the PPh-modified films decreased over time. The number of normal and cancerous osteoblasts seeded on the PPh-modified films was reduced compared to the materials without PPh. However, the reduction was much greater for Saos-2 cells. In addition, when compared with the control PCL film, only PCL/15PPh and PCL-A2mq/15PPh films showed a reduced number of NHOst cells after 5 days of culture, while the other materials with PPh (PCL-A2sg/15PPh and PCL-A2sa/15PPh) showed no significant differences. The number of NHOst and Saos-2 cells cultured on PPh-modified films tended to increase in the following order: PCL/15PPh < PCL-A2mq/15PPh < PCL-A2sg/15PPh < PCL-A2sa/15PPh. In normal osteoblasts, cytotoxicity of the materials decreased over the culture period. The only exceptions were PCL/PPh and PCL-A2mq/15PPh films, which showed no significant changes in cytotoxicity over time. In addition, the cytotoxicity of these materials was the highest after 5 days of culture and was also higher than that of the control PCL material. Other PPh-modified films did not differ from PCL film. The cytotoxicity did not exceed 31% for any of the materials tested. With cytotoxicity up to 19% for the TCPS control and up to 31% for the PCL control material, the films showed no cytotoxic effect on normal osteoblasts. In cancerous osteoblasts, the cytotoxicity of the films without PPh decreased over time, and after 5 days of culture, it was at the same level or even lower than that of the control PCL material. In contrast, in cancerous osteoblasts, the cytotoxicity of the PPh-modified films was significantly higher than that of the films without PPh and increased over time. Among the PPh-containing materials, however, PCL-A2sa/15PPh showed significantly the lowest cytotoxicity.

**Figure 8:**
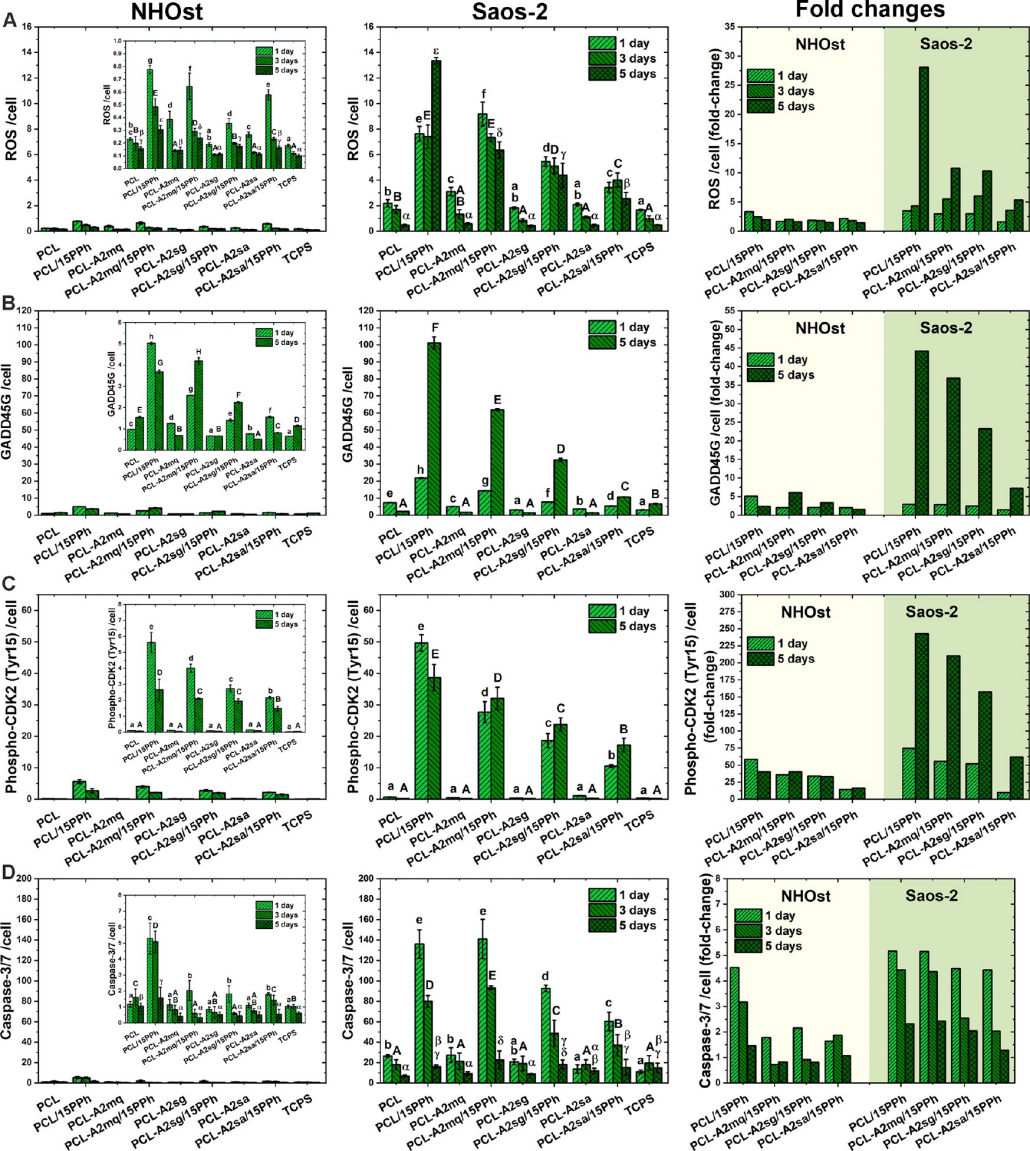
ROS production (A), GADD45G expression (B), phospho-CDK2 (Tyr15) level (C), and caspase-3/7 activity (D) normalized to the number of cells, presented as absolute values (first and second columns) and as fold changes from the material without PPh (third column). Statistically significant differences (*p* < 0.05) between films and TCPS after 1-, 3-, and 5-day cell culture periods are indicated by subsequent lower, upper Latin letters and Greek letters, respectively. Different letters indicate statistically significant differences.

ROS production, GADD45G expression, phospho-CDK2 (Tyr15) level, and caspase-3/7 activity in the normal and cancerous osteoblasts cultured in direct contact with the films, normalized to the number of cells, are shown in Figure 8A–D. The results are expressed as absolute values and as ratios of the means for PPh-modified and unmodified materials (fold changes). The presence of PPh in the materials resulted in a significant increase in all the markers tested, both in normal and cancer cells. In cancerous osteoblasts, however, the increase was generally much higher. After 5 days of culture, ROS production, GADD45 expression, and phospho-CDK2 (Tyr15) level were 5.4- to 28.1-, 7.3- to 44.2-, and 61.6- to 242.7-fold higher in Saos-2 cells, respectively. NHOst showed only 1.4- to 2-, 1.6- to 6.1-, and 16.3- to 40.2-fold increases, respectively. The magnitude of changes in ROS production, GADD45 expression, and phosphorylation of Cdk2 on Tyr15 after 5 days of culture decreased in the following order: PCL/15PPh > PCL-A2mq/15PPh > PCL-A2sg/15PPh > PCL-A2sa/15PPh. In normal osteoblasts, the increase in caspase-3/7 activity on days 1 and 3 was significantly higher for PCL/15PPh material (4.5- and 3.2-fold on days 1 and 3, respectively) compared to other PPh-modified films (1.6- to 2.2- and 0.7- to 1.9-fold). In cancerous osteoblasts, PCL/15PPh (5.2- and 4.4-fold on days 1 and 3, respectively) and PCL-A2mq/15PPh (5.1- and 4.4-fold) films showed significantly higher increases in caspase-3/7 activity on days 1 and 3 compared to PCL-A2sg/15PPh (4.5- and 2.5-fold) and PCL-A2sa/15PPh (4.4- and 2-fold).

The use of agents that lead to excessive production and accumulation of ROS selectively in cancer cells has been shown to be an effective treatment for different cancers. Although the majority of the beneficial effects of polyphenols are attributed to their antioxidant activity, there is evidence that polyphenols may also possess prooxidant properties.^58^ ROS-mediated cancer cell cycle arrest and cell death by apoptosis induced by DNA damage, among others, have been identified as the main molecular pathways responsible for the anticancer activity of polyphenols, also in OS.^59,60^

The PPh-modified films induced significant ROS production while inhibiting proliferation and inducing death (expressed as an increase in the cytotoxicity of the materials) of OS cells. The results suggested that the underlying mechanisms could be ROS-mediated cell cycle arrest and apoptosis. Cdk2 is a master regulator of the G1/S transition and the S phase progression of the cell cycle. Phosphorylation on Tyr15 leads to inactivation of this cyclin.^61^ Therefore, a significant increase in Cdk2 phosphorylation on Tyr15 indicated a G1/S cell cycle arrest in OS cells. GADD45 proteins are important cellular stress sensors and tumor suppressors in genotoxic and nongenotoxic stress responses. Following DNA damage, GADD45 proteins are rapidly induced, leading to cell cycle arrest at both the G1/S and G2/M transitions and apoptosis. They are also actively involved in DNA repair mechanisms. As a result, many drugs have been shown to inhibit cancer cell growth by targeting GADD45 expression.^62,63^ The upregulated expression of GADD45G in Saos-2 cells suggested that PPh-modified films induced cell cycle arrest and apoptosis mediated by the GADD45G pathway. The induction of the caspase-dependent apoptotic pathway, which leads to the cleavage of proteins essential for the survival of the cell and ultimately to its death, is one of the proapoptotic activities of polyphenols in cancer cells.^64^ Significantly increased caspase-3/7 activity indicated that PPh-containing materials induced caspase-mediated apoptosis in OS cells, particularly in the early stages of cell culture. Increasing cytotoxicity with decreasing caspase-3/7 activity over time in Saos-2 cells may suggest that alternative forms of cell death, such as caspase-independent cell death via autophagy, may occur in parallel.^34^ Importantly, the results showed a clear correlation between the PPh-binding capacity of the BGs and the anticancer properties of the films. BGs that bind PPh more effectively reduced the antiproliferative activity and cytotoxicity of PPh-modified films as well as the markers associated with them.

Another important observation was the selective effect of polyphenol-containing materials. They showed a cytotoxic and antiproliferative effect on cancerous osteoblasts but not on normal cells. Similar observations have been made previously for various PPh, including carnosol, carnosic acid,^5^ and curcumin.^65^ Furthermore, Cazzola et al. demonstrated a selective cytotoxic activity of BGs functionalized with gallic acid and natural polyphenols extracted from green tea leaves and red grape skins against OS cells. The presence of the grafted polyphenols increased ROS production and induced DNA damage in the cancer cells, while promoting an anti-inflammatory effect on human fetal preosteoblasts.^7^ Tricalcium phosphate 3D-printed scaffolds loaded with liposomal curcumin prepared by Sarkar and Bose showed significant cytotoxicity against OS cells, while at the same time promoting the viability and proliferation of normal osteoblast cells.^1^

Our previous studies have shown that PCL-A2sg/15PPh composites significantly increased the expression of bone extracellular matrix proteins and alkaline phosphatase activity in normal human osteoblasts, while reducing ROS production in macrophages.^33^ Taking together, the incorporation of polyphenols extracted from sage into PCL/BG composites provided not only potential anticancer activity but also osteogenic and anti-inflammatory properties.

## 4 Conclusions

In this work, BG particles obtained by different methods were used as modifiers of polyphenol-loaded PCL-based composites. The polyphenols extracted from sage improved the hydrophilicity, apatite-forming ability, and mechanical properties of the composites and also provided antioxidant and anticancer activity. As the BG particles had different polyphenol-binding capacities, which resulted from different textural properties (porosity, surface area) and surface chemistry (silanol content), they modulated the kinetics of polyphenol release and the aforementioned properties to a great extent. Importantly, polyphenol-loaded materials exhibited multifaceted and selective anticancer activity, including ROS-mediated cell cycle arrest and apoptosis of OS cells via Cdk2-, GADD45G-, and caspase-3/7-dependent pathways.

Together with their apatite-forming ability, lack of cytotoxicity to normal osteoblasts, as well as osteogenic, and anti-inflammatory properties, the composites are promising biomaterials for use in the treatment of bone defects, particularly after OS tumor resection. The in vivo studies are required to prove and evaluate the osteogenic, anti-inflammatory, and anticancer activity of the materials.

## Supporting Information Available

Results of SEM, EDX, TEM, FTIR, and BET analyses of bioactive glasses (PDF)
