# Supplementary material for: Bioactive Glasses Modulate Anticancer Activity and Other Polyphenol-Related Properties of Polyphenol-Loaded PCL/Bioactive Glass Composites
Source: ACS Appl Mater Interfaces. 2024 May 6;16(19):24261–73. doi: 10.1021/acsami.4c02418 (PMC11103658; doi:10.1021/acsami.4c02418)
Supplement: Supplementary file 1 — am4c02418_si_001.pdf [file am4c02418_si_001.pdf]

Supporting Information

Bioactive glasses modulate anticancer activity and other polyphenol-related properties of polyphenol-loaded PCL/bioactive glass composites

Michal Dziadek[1]*, Kinga Dziadek[2], Kamila Checinska[1], Barbara Zagrajczuk[1], Katarzyna Cholewa-Kowalska[1]**

[1]AGH University of Krakow, Faculty of Materials Science and Ceramics, Department of Glass Technology and Amorphous Coatings, 30 Mickiewicza Ave., 30-059 Krakow, Poland
[2]University of Agriculture in Krakow, Faculty of Food Technology, Department of Human Nutrition and Dietetics, 122 Balicka St., 30-149 Krakow, Poland
Corresponding authors: e-mails: *dziadek@agh.edu.pl; **cholewa@agh.edu.pl

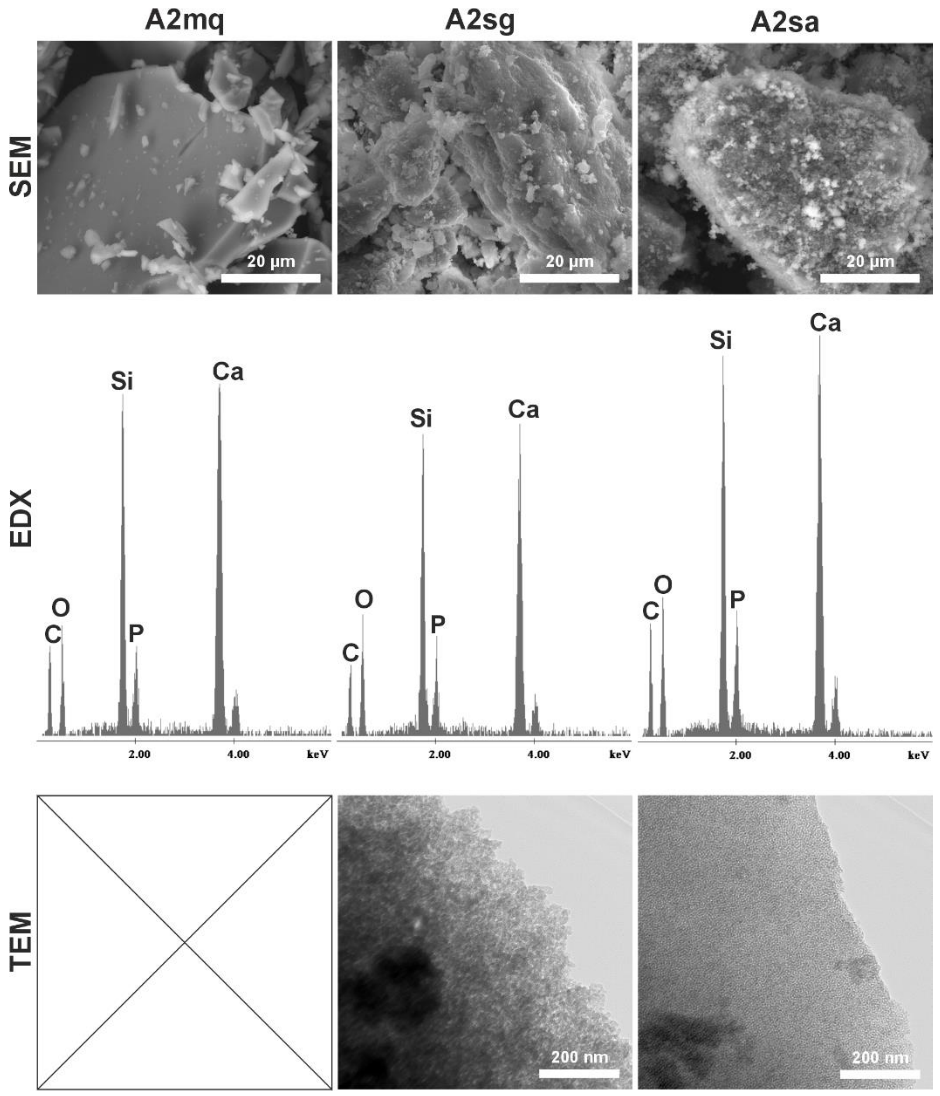

Figure S1. SEM images, EDX spectra (averaged for the entire analysed surface using SEM), and TEM images of the bioactive glasses.

Table S1. Specific surface area, specific pore volume and average pore diameter of the bioactive glasses (measured using BET method).

Bioactive glass	Specific surface area S_{BET} (m^2 g^{-1})	Specific pore volume (cm^3 g^{-1})	Average pore diameter (nm)
A2mq	0.26	-	-
A2sg	62.54	0.21	9.76
A2sa	168.91	0.35	3.59

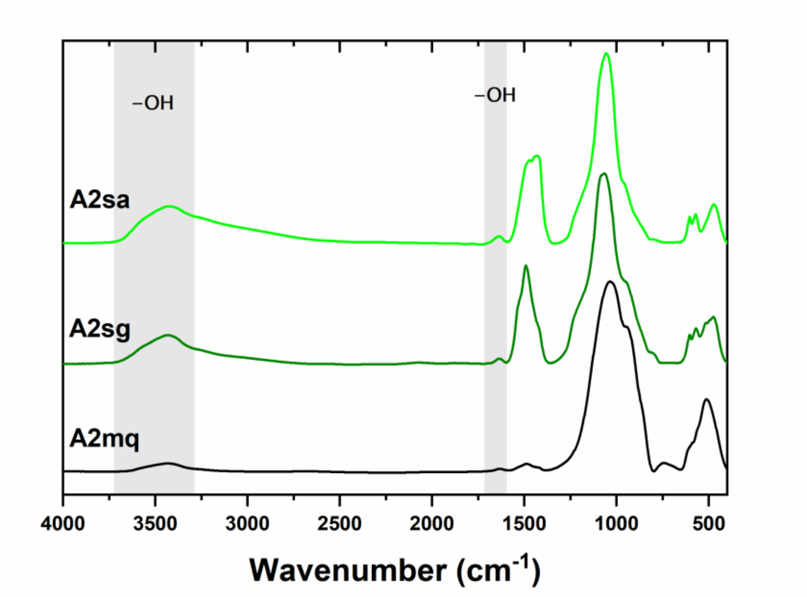

Figure S2. FTIR spectra of the bioactive glasses (collected in transmission mode in KBr pellets).
